# Efficiency and nutritional parameters in an elderly high risk population on hemodialysis and hemodiafiltration in Italy and France: different treatments with similar names?

**DOI:** 10.1186/s12882-018-0948-8

**Published:** 2018-07-09

**Authors:** Giorgina Barbara Piccoli, Gianfranca Cabiddu, Maria Rita Moio, Antioco Fois, Riccardo Cao, Ida Molfino, Ana Kaniassi, Francoise Lippi, Ludivine Froger, Antonello Pani, Marilisa Biolcati

**Affiliations:** 1 Dipartimento di Scienze Cliniche e Biologiche, Università di Torino, Torino, Italy; 2 Nephrologie, Centre Hospitalier Le Mans, Avenue Roubillard 182, 7200 Le Mans, France; 3 Nephrology, Brotzu Hospital, Cagliari, Italy; 4 University of Naples, Naples, Italy; 5 Nutrition Clinique, Centre Hospitalier Le Mans, 7200 Le Mans, France; 6 Obstetrics, Department of Surgery, University of Torino, Torino, Italy

**Keywords:** Hemodialysis, Hemodiafiltration, Albumin, Kt/V, Malnutrition, Elderly, Comorbidity, MIS index, Charlson index

## Abstract

**Background:**

Choice of dialysis is context sensitive, explored for PD and extracorporeal dialysis, but less studied for haemodialysis (HD) and hemodiafiltration (HDF), both widely employed in Italy and France; reasons of choice and differences in prescriptions may impact on dialysis-related variables, particularly relevant in elderly, high-comorbidity patients.

**Methods:**

The study involved two high-comorbidity in-hospital cohorts, treated in Centers with similar characteristics, in Italy (Cagliari) and France (Le Mans). All patients (204) agreed to participate. Stable cases on thrice-weekly dialysis, with at least 2 months follow-up were selected (180 patients, Males 59.4%, median age 71 years, vintage 4.3 years, Charlson index 9). Univariate and multivariate correlations between baseline data, HD-HDF, dialysis efficiency and nutritional markers were assessed.

**Results:**

In Le Mans HDF was mainly chosen to increase efficiency (large surface dialysers, high convective volume; 76.3% of the patients), in Cagliari to improve tolerance (smaller surfaces, lower convective volume; 59% of patients). Kt/V was similar in HD and HDF, and in both settings(median Kt/V Daugirdas 2: 1.6); in the setting of high efficiency no correlation was found between Kt/V, BMI, urea, creatinine, n-PCR and phosphate. The relationship between Kt/V and albumin was divergent: a weak consensual increase was present in Cagliari, a decrease in Le Mans, suggesting a role of albumin losses with high convective volumes. In the multivariate analysis, after adjustment for other covariates (including comorbidity and type of treatment) low albumin level < 3.5 g/dl was highly correlated with setting of study: Le Mans (OR: 7.155 (2.955–17.324)). The multivariate analysis confirmed a role of type of treatment, with higher risk of low albumin levels in HDF (OR: 3.592 (1.466–8.801)), and of comorbidity (Charlson index> = 7 (OR: 3.153 (1.311–7.582)), MIS index> = 7 (OR: 5.916 (2.457–14.241)).

**Conclusions:**

The different prescriptions of HD and HDF may have similar effects on dialysis efficiency, but diverging effects on crucial nutritional markers, such as albumin levels, probably more evident in high-comorbidity populations.

## Background

The choice of dialysis is context sensitive, and while the focus is usually on the treatment choice (peritoneal dialysis versus extracorporeal dialysis, hemodialysis versus hemodiafiltration), it is also acknowledged that results do not only depend upon the treatment in itself, but upon subtle differences in prescriptions and modalities. Indeed, this issue has been extensively explored for PD and extracorporeal dialysis, but less studied for haemodialysis (HD) and hemodiafiltration (HDF) [1–5].

HF and HDF are widely employed in several European Countries, including Italy and France; both treatments may be differently prescribed, and for different goals, thus impacting on dialysis-related variables; the impact may be higher in elderly, high-comorbidity patients, with high mortality at baseline [6–8]. Differences in prescriptions, and not only in methods, may be at the basis of the inconsistent results of comparisons between modes of treatment, available in the literature [9, 10].

Hence, we undertook this cross-sectional study aimed at exploring in detail the relationship between dialysis prescriptions, selection criteria, dialysis efficiency and nutritional parameters in two relatively large cohorts of high-comorbidity dialysis patients, treated with haemodialysis (HD) and hemodiafiltration (HDF) in Italy and France.

The two centers where the study was undertaken (Cagliari in Italy and Le Mans in France) are similar in a number of ways: they are situated in relatively small cities, with large, mainly rural, areas of referral, work in cooperation with networks of out-of-hospital facilities, and share a policy of selecting the patients with the highest comorbidity for in-hospital dialysis. Their policies on selecting patients for HD and HDF are quite different, however. While in Italy the criterion driving the choice of HDF is its superior tolerance, in France it is its efficiency [9, 11].

It is hoped that the present study will help in the interpretation of commonly used clinical markers in patients with high comorbidity, treated by HDF or HD, favouring comparison between dialysis policies, and ultimately casting light on the specific influences of different prescriptions.

## Methods

### Settings and patients: Le Mans

The Centre Hospitalier du Mans (CHM), which is presently one of the three largest non- university hospitals in France, has about 1700 beds, 500 in geriatric units and about 300 for different medical specialties (at the time of the study, 20 are in the nephrology unit).

Le Mans has about 150,000 inhabitants, with approximately another 150,000 people living in its suburbs; CHM serves a population of about 800,000. Two dialysis facilities run by a non-profit association (ECHO) are present in the area but nephrology beds are only available at CHM.

The dialysis ward has 25 beds. These are occupied by chronic dialysis patients, by patients with AKI (not needing intensive care) as well as by patients from the out-of hospital centers run by the ECHO who need to be hospitalized. The pool of patients on chronic treatment ranges from 95 to 110, depending on the incidence of kidney transplantation, death and transfers. In keeping with the indications of the French Society of Nephrology (Société Francophone de Néphrologie, Dialyse et Transplantation, SFNDT [12]), out-of-hospital dialysis is widely used and only cases posing particular clinical, attitudinal or psychological problems are managed in the hospital. Hence, the population studied is a large sample of the most difficult patients treated in the area. The ratio between cases treated at CHM and those treated at ECHO is around 1:6.

### Settings and patients: Cagliari

The study was conducted at Ospedale Brotzu, which is currently the largest hospital in Sardinia, Italy’s largest island. The hospital has about 600 beds (22 in the nephrology unit) and a transplant center, performing about 50 kidney grafts per year. Cagliari has a population of about 150,000 inhabitants, which rises to 250,000 if those living in districts surrounding the city are counted, and the entire area Ospedale Brotzu serves has a population of about 600,000. Nephrology beds are only available in the hospital. A network of public out-of-hospital dialysis facilities provides treatment for patients with lower comorbidity, in keeping with Italian experiences [13]. The ratio between patients treated at Ospedale Brotzu and those receiving dialysis in out-of hospital settings is around 1:6.

The dialysis unit has 26 beds dedicated to chronic patients and 8 for patients with acute kidney injury (AKI) or chronic kidney disease (CKD), from other Units, requiring hospitalization. The pool of patients on chronic treatment ranges from 90 to 100, depending up kidney transplantation, death and transfers. Between 45 and 50 peritoneal dialysis patients are also followed.

### Selection criteria

Data were recorded for all patients, but the comparative analysis considered only the cases receiving dialysis 3 times a week, excluding patients on less or more frequent treatments, those with particular indications (for example cardiorenal syndrome), those recovering renal function, or with a life expectancy of 1 month or less. Data of the excluded patients are available in table Appendix-1.

### Dialysis schedules

Le Mans: the most widely-used technique was post-dilutional on-line hemodiafiltration (HDF), with acetate-free citrate-based dialysate (calcium concentration 1.5–1.75 mmol/L; Sodium 138–140 mEq/L, Bicarbonate 32–38, Temperature 36–37 °C; Potassium: 2 mmol/L corrected with potassium infusion in case of need). At time of study, the prescription followed traditional French guidelines: high permeability, a surface at least as wide as the patient’s body surface, reinfusion of at least 24 l per session [14, 15]. Conventional hemodialysis was performed with the same dialysate, employing both medium-low and high permeability dialysers, once more in keeping with the French policy of a large surface and high-efficiency dialysers. About two thirds of the patients were dialysed with an arterovenous fistula (AV fistula), the rest with a permanent tunnelised catheter.

Cagliari: the most widely used technique was hemodialysis (HD) with bicarbonate-based, acetate-containing dialysate. About 40% of the patients are on on-line hemodiafiltration (HDF), 85% post-diluitional. On both techniques, the dialysate used is composed of Sodium 140 mEq/L; Bicarbonate 32 mmol/L, Calcium 1.5 mmol/L, T 36.5 °C, Potassium 2–3 mmmol/L. Polysulfone, high-permeability dialysers are employed in HD and HDF. Since in Italy the choice of HDF is aimed principally at improving tolerance and ensuring hemodynamic stability, the main surfaces employed were smaller (1.4 and 1.7 m2), and reinfusion volumes lower (10 to 16 l per session). Vascular access was an AV fistula for about 80% of the patients and a permanent catheter for the remaining 20%.

### Control policies and database

The following general information was gathered for all cases, in both centers: age, sex, end-stage kidney disease, vintage of renal replacement therapy and dialysis, educational level, present and past work, marital status, other members of household, previous kidney graft or waitlist for transplantation.

For the sake of the present study, we considered the data available at the update at the comorbidity and nutritional assessment (Le Mans, July 2016; Cagliari, December 2016) integrated with the information from a previous or subsequent update, if the patient missed the appointment, or there were acute clinical problems (for example a febrile illness). Only the patients with at least 2 months of treatment were included.

The following data, routinely employed in both Centers, were gathered: creatinine (mg/dL), urea before and after dialysis (mg/dL), calcium (mmol/L), phosphore (mmol/L), PTH (pg/ml), 25-OH vitamin D3 (ng/ml), Bicarbonate (mmol/L), albumin (g/L), total protein (g/L), transferrin (g/L), ferritin (micrograms/L), hemoglobin (g/dl), C-reactive protein (CRP) (mg/dl), total cholesterol (mg/dl), Brain natriuretic peptide (BNP) (pg/ml), glycated hemoglobin (%).

Dialysis efficiency was calculated using Daugirdas II Kt/V, and the patient’s normalized protein catabolic rate (nPCR) was assessed using the two-point formula; however, in discrepant cases (clinical data suggesting malnutrition, and calculation indicating a high PCR), the three-point formula was also calculated, and the vascular access was examined for evidence of recirculation.

Comorbidity was assessed using the Charlson Index; to avoid colinearity, the corrected formula suggested for dialysis patients, that also considers albumin levels, was not employed in this analysis [16, 17].

Two internationally validated questionnaires were chosen for nutritional assessment: the Subjective Global Assessment (SGA) and Malnutrition Inflammation Score (MIS) [18, 19].

### Statistical analysis

A descriptive analysis was performed as appropriate (mean and standard deviation for parametric data and median and range for non-parametric data). Independent t-test, Chi-square test, Fisher’s test, Mann-Whitney U test were used, where indicated, for comparisons between groups. The Anova Test and Kruskal-Wallis Tests were used for discrete variables. Significance was set at < 0.05.

A logistic regression analysis was performed between albumin level and cholesterol level, Kt/V, nPCR, MIS, age, and Charlson Index. All data were analysed as continuous parameters. Data were stratified by setting (Cagliari/Le Mans) and type of dialysis (HD/HDF).

A multivariate analysis considered albumin level dichotomised at < 3.5 g/dL as the outcome and the previous parameters as covariates, dichotomised at the median or at a clinically relevant target level, if near the median (Kt/V: 1.5, considering adequate dialysis; nPCR: 1 g/Kg/day; cholesterol 200 mg/dL, considered as the upper normal value, and near the median of our population; Charlson Index 7, usually considered as the limit between high and low comorbidity; MIS 7, near the median level observed in our population). The analysis was performed with SPSS software (version 24.0).

### Ethical issues

In both centers all the patients treated were adults; all patients agreed to allow use of their routine clinical and biochemical data for the sake of this study. Written consent was gathered from all patients, and included consent for data collection and publication; in the case of language barriers or of intellectual deficits, the patient’s proxy or legal guardian was asked for permission.

Since the present cross sectional study did not involve additional blood tests or the use of imaging techniques, no ethics committee approval was needed in Italy; in keeping with current French legislation, the study was approved by the ethics committee in Le Mans (“Avis favorable du groupe d’éthique du Centre Hospitalier du Mans”, 16 mars 2017).

## Results

### Overall data

Table 1 reports the main baseline data of the patients treated in the two settings.

**Table 1:** Overall data of the study population

	Study population	Study population Le Mans	Study population Cagliari	P Le Mans vs Cagliari
N	180	97	83	–
Males/Females	59.4% 40.6%	55.7% 44.3%	63.9% 36.1%	0.336
Age median (yrs) (min-max)	69 (18–90)	71 (18–90)	67 (26–89)	0.020
RRT vintage (yrs) (min-max)	6.7 (0.07–43.5)	4.3 (0.07–43.5)	10.3 (0.5–36.9)	< 0.001
Charlson median (min-max)	8 (2–16)	9 (2–16)	6 (2–12)	< 0.001
MIS median (min-max)	7 (1–27)	7 (1–23)	6 (2–27)	0.020
SGA: A	61.1%	49.5%	74.7%	0.002
SGA: B	33.9%	45.4%	20.5%	
SGA: C	5.0%	5.2%	4.8%	
HD-HDF (%)	HD: 40.0% HDF: 60.0%	HD: 23.7% HDF: 76.3%	HD: 59.0% HDF: 41.0%	< 0.001
BMI Kg/m2 median (min-max)	24.3 (14.6–47.1)	26.2 (16.4–47.1)	22.6 (14.6–31.8)	< 0.001
BMI < 20	32 (18.0%)	11 (11.6%)	21 (25.3%)	< 0.001
BMI 20–25	66 (37.1%)	26 (27.4%)	40 (48.2%)	
BMI 25–30	56 (31.5%)	38 (40.0%)	18 (21.7%)	
BMI > = 30	24 (13.5%)	20 (21.1%)	4 (4.8%)	
Albumin (g/dl) median (min-max)	3.4 (2.0–4.5)	3.2 (2.6–3.8)	3.7 (2.0–4.5)	< 0.001
Albumin < 3	23 (12.8%)	22 (22.7%)	1 (1.2%)	< 0.001
Albumin < 3.5	99 (55.0%)	78 (80.4%)	21 (25.3%)	< 0.001
BUN (mg/dl) median (min-max)	60.0 (24.4–105)	56.7 (24.4–100)	63.0 (32.0–105)	0.015
Creatinine (mg/dl) median (min-max)	9.1 (2.7–15.0)	8.8 (2.7–15.0)	10.2 (3.6–14.8)	0.003
Cholesterol (mg/dl) median (min-max)	158 (49–263)	162.8 (49–249)	157 (67–263)	0.978
Kt/V median (min-max)	1.6 (0.7–2.3)	1.5 (0.7–2.2)	1.6 (0.8–2.3)	0.005
nPCR (g/Kg/day) median (min-max)	1.04 (0.5–1.8)	0.9 (0.5–1.7)	1.1 (0.6–1.8)	0.002

Legend: *RRT* renal replacemet therapy, *MIS* Malnutrition inflammation score, *SGA* subjective global assessment (A. well nourished, B moderate malnutrition, C severe malnutrition), *HD* hemodialysis, *HDF* hemodiafiltration, *BUN* blood urea nitrogen (predialysis), *Kt/V* dialysis efficiency index, according to Daugirdas 2 formula, *nPCR* normalised protein catabolic rate

The selection of patients on thrice-weekly dialysis, with a life expectancy of at least one month, without alimentary disorders, including those associated with dementia, excluded 27 patients. Among them were 4 on once-weekly dialysis, 3 on twice-weekly dialysis, 2 in the phase of recovery of kidney function (several months after dialysis start), 14 for very low life expectancy (terminal neoplasia 3 cases; cardiovascular disease in the others, often with dementia) (Appendix).

There were some relevant baseline differences in the two populations. The patients in Cagliari, Italy were younger (67 vs 71 years of age), had a lower BMI (22.6 vs 26.2), but had a significantly longer treatment vintage (10.3 vs 4.3 years). The Charlson Index, that integrates age, is significantly higher for Le Mans (9 vs 6) (Table 1). MIS and cholesterol levels were not significantly different, but BMI was higher and SGA lower in Le Mans. Conversely, pre-dialysis Blood urea nitrogen (BUN), Kt/V (Daugirdas II) and nPCR were higher in Cagliari than in Le Mans.

While both centers offer hemodialysis (HD) and hemodiafiltration (HDF), HD was the treatment more frequently chosen in Cagliari, while HDF was the one more widely employed in Le Mans.

### Treatment choice: HD versus HDF

Tables 2 and 3 report the main characteristics of the patients sorted according to the type of dialysis they received (HD or HDF) and to study setting.

**Table 2:** The main dialysis parameters of the study population, according to mode of dialysis (HD versus HDF) and setting of study

	HDF Le Mans	HDF Cagliari	HDF all cases	P Le Mans vs Cagliari HDF	HD Le Mans	HD Cagliari	HD all cases	P Le Mans vs Cagliari HD
N	74	34	108	–	23	49	72	–
Males/Females	54.1% 45.9%	70.6% 29.4%	59.3% 40.7%	0.104	60.9% 39.1%	59.2% 40.8%	59.7% 40.3%	0.892
Age median (min-max)	69 (31–87)	68.5 (41–87)	69 (31–87)	0.608	78 (18–90)	65 (26–89)	70 (18–90)	0.001
RRT vintage (min-max)	5.2 (0.2–43.5)	9.8 (0.6–34.9)	5.9 (0.2–43.5)	0.001	2.7 (0.1–14.3)	10.8 (0.5–36.9)	7.9 (0.1–36.9)	< 0.001
KT/V Daugirdas median (min-max)	1.6 (0.7–2.2)	1.7 (0.8–2.3)	1.6 (0.7–2.3)	0.013	1.5 (1.2–1.9)	1.6 (0.9–2.3)	1.6 (0.9–2.3)	0.104
nPCR median (min-max)	0.9 (0.5–1.7)	1.1 (0.6–1.5)	0.9 (0.5–1.7)	0.138	1.0 (0.7–1.3)	1.1 (0.6–1.8)	1.1 (0.6–1.8)	0.046
AV fistula, N (%)	61 (82.4%)	27 (79.4%)	88 (81.5%)	0.913	13 (56.5%)	40 (81.6%)	53 (73.6%)	0.049
Hours of dialysis (^a^)	4 (3–4.5)	4 (3–4.5)	4 (3–4.5)	0.729	4 (2.5–4.0)	4 (3–4.5)	4 (2.5–4.5)	0.942
Intradialytic Weight loss	1.7 (0.1–3.9)	2.2 (0.2–3.1)	2.0 (0.1–3.9)	0.148	1.2 (0.3–3.7)	2.1 (0.9–4.1)	1.9 (0.3–4.1)	0.011

*Legend M: RRT* Renal replacemet therapy, *HD* Hemodialysis, *HDF H*emodiafiltration, *BUN* Blood urea nitrogen (predialysis), *Kt/V* Dialysis efficiency index, according to Daugirdas 2 formula, *nPCR* Normalised protein catabolic rate, *AV* Fistula: arteriovenous fistula

Note^a^: only cases treated 3 times per week were selected

**Table 3:** Other biochemical and nutritional parameters in the study population according to mode of dialysis (HD versus HDF) and setting of study

	HDF all cases	HDF Le Mans	HDF Cagliari	P Le Mans vs Cagliari HDF	HD Le Mans	HD Cagliari	HD all cases	P Le Mans vs Cagliari HD
N	108	74	34	–	23	49	72	–
Charlson median (min-max)	8 (2–16)	9 (2–16)	6 (3–11)	< 0.001	9 (2–15)	6 (2–12)	7 (2–15)	< 0.001
MIS median (min-max)	6.5 (1–27)	7 (1–23)	6 (2–27)	0.412	8 (5–19)	6 (2–18)	7 (2–19)	0.005
SGA: A	62 (57.4%)	37 (50.0%)	25 (73.5%)	0.053	11 (47.8%)	37 (75.5%)	48 (66.7%)	0.054
SGA: B	40 (37.0%)	33 (44.6%)	7 (20.6%)		11 (47.8%)	10 (20.4%)	21 (29.2%)	
SGA: C	6 (5.6%)	4 (5.4%)	2 (5.9%)		1 (4.3%)	2 (4.1%)	3 (4.2%)	
BMI Kg/m2 median (min-max)	24.8 (14.6–47.1)	26.1 (18.2–47.1)	22.7 (14.6–30.0)	< 0.001	27.0 (16.4–31.2)	22.6 (16.1–31.8)	23.7 (16.1–31.8)	0.004
Albumin (g/dl) median (min-max)	3.3 (2.0–4.3)	3.2 (2.6–3.8)	3.5 (2.0–4.3)	< 0.001	3.2 (2.7–3.8)	3.9 (3.0–4.5)	3.7 (2.7–4.5)	< 0.001
Cholesterol (mg/dl) median (min-max)	160.0 (49.5–241.3)	166.3 (49.5–241.3)	157.5 (67.0–198.0)	0.434	160.1 (98.2–248.6)	153.0 (85.0–263.0)	157.8 (85.0–263.0)	0.970
Hb (g/dL) median (min-max)	11.3 (8.0–14.9)	11.7 (8.4–14.9)	10.8 (8.0–13.4)	0.008	11.3 (10.1–14.9)	11.2 (8.3–13.1)	11.2 (8.3–14.9)	0.234
CRP (mg/dL) median (min-max)	0.6 (0.2–9.4)	0.6 (0.2–9.4)	0.5 (0.3–8.6)	0.219	0.5 (0.4–9.8)	0.5 (0.3–5.1)	0.5 (0.3–9.8)	0.069
PTH (pg/ml) median (min-max)	312 (3–2144)	354 (3–2144)	156.5 (3.7–1082)	0.002	329 (84–1655)	255.5 (3.7–1536)	277 (3.7–1655)	0.062
BNP (pg/ml) median (min-max)	236.5 (7–2950)	252.5 (7–2950)	221.5 (14–2242)	0.238	296 (51–2574)	206 (28–1101)	254.5 (28–2574)	0.082

Legend *M* males, *F* females, *MIS* Malnutrition inflammation score, *SGA* subjective global assessment (A. well nourished, B moderate malnutrition, C severe malnutrition), *HD* hemodialysis, *HDF* hemodiafiltration, *BMI* body mass index, *Hb* haemoglobin, *CRP* C reactive protein, *PTH* parathyroid hormone, *BNP* blood natriuretic peptide

In Le Mans HD is primarily chosen for older patients (an average of almost 10 years older than patients on HDF), with higher MIS (8 versus 7), in the context of a very high comorbidity burden (Charlson index 9 in both cases); conversely, in Cagliari age is higher on HDF (68.5 versus 65 years) while in both settings the other measured differences between the HD and HDF populations are mainly non-significant (Tables 2-3).

Overall, in both settings, Kt/V is at or above target (overall 1.6); predialysis BUN and creatinine are higher in Cagliari, in spite of a lower average BMI; nPCR is lower in Le Mans, but in both settings the levels are lower than the 1.2 g/Kg/day considered as the “ideal” target for the overall dialysis population (Tables 1, 2, 3).

### Relationship between albumin levels and other commonly used nutritional markers

**Fig. 1:**
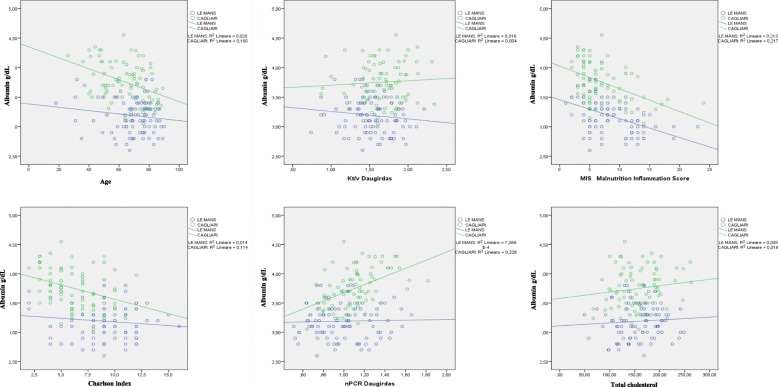
Relationship between serum albumin and other nutritional markers. Note: HD and HDF: statistical significance reached for: Albumin and n PCR: Cagliari R2 0.228 = r 0.478 *P* < 0.01; Albumin and Charlson index: Cagliari R2 0.114 = r 0.337 *P* < 0.01; Albumin and MIS: Le Mans R2 0.213 = r 0.461 *P* < 0.01; Cagliari R2 0.217 = r 0.466 *P* < 0.01; Albumin and age: Cagliari R2 0.166 = r 0.407 *P* < 0.01

**Fig. 2:**
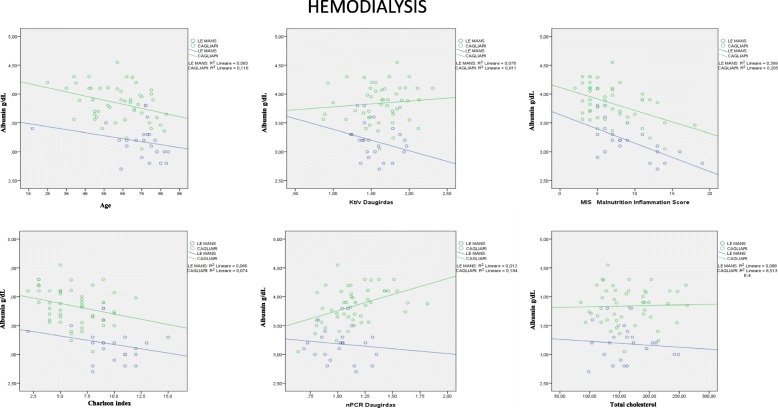
Relationship between serum albumin and other nutritional markers: patients on hemodialysis. Note: HD: statistical significance reached for: Albumin and nPCR: Cagliari R2 0.184 = r 0.429 *P* < 0.01; Albumin and MIS: Le Mans R2 0.399 = r 0.632 *P* < 0.01; Cagliari R2 0.205 = r 0.452 *P* < 0.01; Albumin and age: Cagliari R2 0.116 = r 0.341 *P* < 0.05

**Fig. 3:**
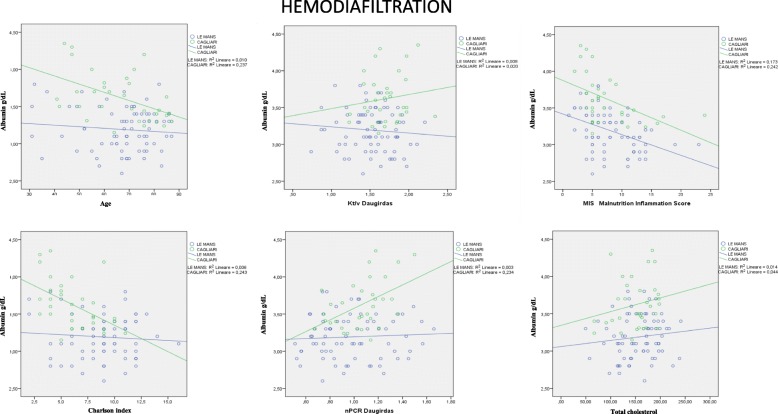
Relationship between serum albumin and other nutritional markers: patients on hemodiafiltration. Note: HDF: statistical significance reached for: Albumin and nPCR: Cagliari R2 0.234 = r 0.484 *P* < 0.01; Albumin and Charlson index: Cagliari R2 0.243 = r 0.493 *P* < 0.01; Albumin and MIS: Le Mans R2 0.173 = r 0.416 *P* < 0.01; Cagliari R2 0.242 = r 0.492 *P* < 0.01; Albumin and age: Cagliari R2 0.237 = r 0.487 *P* < 0.01

Figures 1, 2, 3 report on the association between albumin levels and other relevant nutritional parameters in the overall cohort (Fig. 1), and according to the treatment chosen (Fig. 2: HD, Fig. 3 HDF). The “best fitting” linear curve is designed for each setting.

There is no significant relationship between albumin levels, cholesterol and Kt/V in either setting. Conversely, the relationship between albumin, nPCR, Charlson Index and age is significant in Cagliari but not in Le Mans, while the only significant relationship observed in both places is the link between MIS and albumin levels (*p* < 001) (Fig. 1). The figures are similar on HD and HDF (Figs. 2, 3).

No significant relationship was also found between albumin levels, BMI, predialysis urea, creatinine, and phosphate; conversely, a trend towards lower albumin levels throughout SGA classes was found (Cagliari: albumin level: SGA A: 3.8 (3.1–4.5); B: 3.4 (3.0–4.0); C: 3.4 (2.0–3.8); Le Mans: SGA A: 3.2 (2.6–3.8); B: 3.1 (2.7–3.8); C: 3.0 (2.8–3.0) *p* = 0.01 across SGA stages, and *p* < 0.001 between Cagliari and Le Mans).

Interestingly, the relationship between Kt/V and albumin is divergent in Cagliari (statistically significant consensual increase) and in Le Mans, where a paradoxical, albeit non significant decrease in albumin levels is observed with increasing Kt/V; similarly, the significant increase in albumin we expected would correlate with nPCR was observed only in Cagliari, while on the contrary the line was flat in Le Mans.

### Multivariate analysis

The multivariate analysis, shown in Table 4, shows a different pattern in the two settings: in both Charlson Index above 7 and MIS above 7 correlated with an albumin level below 3.5 g/dl in the univariate analysis, but the significance is retained only in Le Mans for the multivariate analysis. Conversely, HDF reached statistical significance in the univariate and multivariate analysis only in Cagliari.

**Table 4:** Univariate and multivariate logistic regression analysis in each setting of study: outcome: albumin level < 3.5 g/dL

	UNIVARIATE OR (CI 95%)	MULTIVARIATE OR (CI 95%)
CAGLIARI (83 patients)		
HD	1	1
HDF	**5.658** **(1.902–16.826)**	**8.683** **(2.349–32.101)**
KT/V Daugirdas < 1.5	1	1
KT/V Daugirdas> = 1.5	0.944 (0.315–2.834)	0.762 (0.197–2.945)
nPCR Daugirdas < 1	1	1
nPCR Daugirdas > = 1	**0.239 (0.084–0.678)**	0.366 (0.096–1.390)
Cholesterol < 150 mg/dL	1	1
Cholesterol > = 150 mg/dL	0.849 (0.314–2.290)	1.076 (0.300–3.858)
Charlson < 7	1	1
Charlson > = 7	**2.955 (1.062–8.217)**	3.094 (0.826–11.591)
MIS < 7	1	1
MIS > = 7	**4.881 (1.653–14.417)**	3.651 (0.891–14.962)
LE MANS (97 patients)		
HD	1	1
HDF	0.828 (0.245–2.799)	1.225 (0.293–5.126)
KT/V Daugirdas < 1.5	1	1
KT/V Daugirdas > = 1.5	1.200 (0.438–3.286)	1.822 (0.523–6.351)
nPCR Daugirdas < 1	1	1
nPCR Daugirdas > = 1	0.733 (0.268–2.001)	0.585 (0.159–2.151)
Cholesterol < 150 mg/dL	1	1
Cholesterol > = 150 mg/dL	1.056 (0.381–2.925)	0.975 (0.305–3.114)
Charlson < 7	1	1
Charlson > = 7	**4.945 (1.602–15.263)**	**4.285 (1.129–16.263)**
MIS < 7	1	1
MIS > = 7	**7.500 (2.261–24.881)**	**8.451 (2.292–31.161)**

Legend *M* males, *F* females, *MIS* Malnutrition inflammation score, *SGA* subjective global assessment (A. well nourished, B moderate malnutrition, C severe malnutrition), *HD* hemodialysis, *HDF* hemodiafiltration, *BMI* body mass index, *Hb* haemoglobin, *CRP* C reactive protein, *PTH* parathyroid hormone, *BNP* blood natriuretic peptide

Data in bold are statistically significant

When the two settings are combined, low albumin levels are highly correlated with MIS, Charlson index and HDF; however, the highest correlation regards setting of study, suggesting differences not captured by these “macro” definitions.

## Discussion

The dialysis population is getting older and the complexity of the patients is increasing. In particular in settings such as Italy and France, in which an efficient out-of-hospital network provides treatment for patients with lower comorbidity, in-hospital centers follow selection of the most “difficult cases” [12, 13]. Dialysis choice, and prescription modulation is obviously crucial; however, most parameters on which dialysis prescriptions are modulated are standardised in younger populations, and in HD, and less is known on the behaviour of the same parameters in negatively selected cohorts, treated by different dialysis modalities, such as HDF.

The complexity of the study cohorts is indicated by an overall median Charlson Index of 8, corresponding to an expected survival rate of around 30% over 2 years; in the context of high cormorbidity, age and Charlson index were higher in Le Mans (9 in Le Mans versus and 6 in Cagliari), vintage of renal replacement therapy, a further important survival marker, was higher in Cagliari (10.3 years in Cagliari vs 4.3 years in Le Mans) (Tables 1, 2, 3) [20, 21].

There are two main result of our study of potential clinical relevance.

The first point is that, at difference with what has been described in large, non selected dialysis population, mainly on HD, in our high-comorbidity, elderly dialysis populations, with high dialysis efficiency (median Daugirdas 2 Kt/V: 1.6) the relationship between the different markers of comorbidity, nutrition and dialysis efficiency is not a close one [22–25]. In particular neither Kt/V, nor albumin levels were strictly correlated with BMI, cholesterol levels and n-PCR. Furthermore, we did not find a correlation between dialysis efficiency, MIS or SGA and Charlson index, while only the composite MIS was highly correlated with albumin levels (Figs. 1, 2, 3). The correlation is probably not explained by colinearity (MIS integrates albumin, but albumin level accounts for only 10% of the malnutrition inflammation score).

The second point regards the two main survival and treatment markers, Kt/V (efficiency) and albumin (nutrition). In our study, their relationship was context sensitive: Kt/V shows a significant direct correlation with albumin level in Cagliari, and a non-significant inverse correlation in Le Mans (Figs. 1, 2, 3). Interestingly, no correlation was found between nPCR and albumin level in Le Mans while a direct correlation was found in Cagliari. This pattern strongly suggests that differences in prescriptions have a nearly opposite effect on albumin level. Since HDF in Le Mans is performed with large dialysers and high convective volume, the inverse relationship with Kt/V supports a crucial role of albumin loss. Albumin loss is a well-known effect of HDF, but its clinical role is often considered as minor, while our study suggests that the effect be highly relevant in the elderly dialysis population. Quantification of albumin loss by direct analysis of the dialysate, together with analysis of markers of hepatic synthesis, may be a next step to guide dialysis choice avoiding hypoalbuminemia [26–31].

The multivariate analysis confirms the association of patient-related measures (Charlson Index, MIS) with albumin levels, dichotomised at 3.5 g/dl, and the odds ratio of low albumin level is significantly higher for HDF, in keeping with the interpretation of a central role for albumin loss (Tables 4-5). The association between the study setting and albumin levels is the most significant one in the multivariate analysis, which adjusted for all the previous covariates. This suggests that merely identifying the treatment used (HD or HDF) is not sufficient and that profoundly different treatments are grouped under the same label (Table 5).

**Table 5:** Univariate and multivariate logistic regression analysis: outcome: albumin level < 3.5 g/dL

	UNIVARIATE OR (CI 95%)	MULTIVARIATE OR (CI 95%)
HD	1	1
HDF	4.092 (2.173–7.703)	**3.592 (1.466–8.801)**
KT/V Daugirdas < 1.5	1	1
KT/V Daugirdas> = 1.5	0.715 (0.386–1.326)	1.131 (0.473–2.703)
nPCR Daugirdas < 1	1	1
nPCR Daugirdas > = 1	0.337 (0.181–0.626)	0.552 (0.240–1.272)
Cholesterol < 150 mg/dL	1	1
Cholesterol > = 150 mg/dL	1.039 (0.572–1.887)	1.030 (0.451–2.350)
Charlson < 7	1	1
Charlson > = 7	6.545 (3.329–12.871)	**3.153 (1.311–7.582)**
MIS < 7	1	1
MIS > = 7	4.690 (2.492–8.825)	**5.916 (2.457–14.241)**
CAGLIARI	1	1
LE MANS	12.120 (5.992–24.517)	**7.155 (2.955–17.324)**

Legend *HD* hemodialysis, *HDF* hemodiafiltration, *MIS* Malnutrition inflammation score, *Kt/V* dialysis efficiency index, according to Daugirdas 2 formula, *nPCR* normalised protein catabolic rate, *Charlson* Charlson index

Data in bold are statistically significant

The limits of this study are many. The populations, although studied in detail, were relatively small; we studied the relationship between parameters assessed at a single time, while trajectories are probably more sensitive outcome markers.

While the multivariate approach accounts, at least partially, for the baseline differences, the populations were not homogeneous, thus creating the risk of colinearity in some measures; in particular, the population in Cagliari was younger, with higher prevalence of AV fistula; the higher predialysis BUN and creatinine in Cagliari may the expression of a better preserved nutritional state, not completely accounted for by the stratification for age. Furthermore, as cited, some degree of colinearity links albumin and MIS, although albumin accounts for only 10% of MIS score.

The decision to consider only inexpensive, routine markers, means that other important markers or analyses, such as prealbumin or bioimpedance analysis, not yet routinely used in clinical practice, were not considered, but will be required to interpret our data.

These limitations stress the need to interpret with caution the usual nutritional markers in high-risk dialysis patients [32].

Indeed, the relationship between the most studied marker of nutrition, serum albumin, and the other markers of dialysis care may be less strict than previously described, at least in high comorbidity populations and the dialysis technique may interfere with results.

Our study therefore suggests that we need to further refine our knowledge by studying cohorts stratified according to a detailed dialysis prescription, including more markers and analyses, so that the roles played by patients’ innate characteristics and treatment-related components can be more clearly discerned.

## Conclusions

In elderly, high comorbidity dialysis population, treated by HD and HDF, and in which dialysis efficiency target is met, the correlations among the classic efficiency and nutrition markers, well described in younger populations on HD, is weak or absent. After correction for comorbidity and treatment, the setting of study was significantly related to albumin levels; this sensitivity to context emphasizes the importance of subtle differences in dialysis prescriptions, indirectly suggesting that fine modulation of dialysis schedules may be a tool for controlling albumin levels, and perhaps improving nutritional status, in fragile dialysis patients.

## Appendix

**Table 6:** Excluded and included cases

	Overall cohort	Excluded cases	Study population
N	207	27	180
Males/Females	59.4% 40.6%	59.3% 40.7%	59.4% 40.6%
Age median (yrs) (min-max)	69 (18–90)	67 (32–90)	69 (18–90)
RRT vintage (yrs) (min-max)	6.4 (0.2–43.5)	3.2 (0.2–24.6)	6.7 (0.07–43.5)
Charlson median (min-max)	8 (2–16)	7 (2–14)	8 (2–16)
MIS median (min-max)	7 (1–27)	9.5 (1–27)	7 (1–27)
SGA: A	58.0%	37.0%	61.1%
B	32.9%	25.9%	33.9%
C	6.8%	18.5%	5.0%
HD-HDF (%)	HD: 33.9% HDF: 66.1%	HD: 59.3% HDF: 40.7%	HD: 40.0% HDF: 60.0%
BMI Kg/m2 median (min-max)	24.2 (14.6–47.1)	22.8 (16.6–43.4)	24.3(14.6–47.1)
BMI < 20	41 (20.0%)	9 (33.3%)	32 (18.0%)
BMI 20–25	75 (36.6%)	9 (33.3%)	66 (37.1%)
BMI 25–30	62 (30.2%)	6 (22.2%)	56 (31.5%)
BMI > = 30	27 (13.2%)	3 (11.1%)	24 (13.5%)
Albumin (g/dl) median (min-max)	3.4 (1.9–4.5)	3.4 (1.9–4.1)	3.4 (2.0–4.5)
Albumin < 3	28 (13.6%)	5 (19.2%)	23 (12.8%)
Albumin < 3.5	114 (55.3%)	15 (57.7%)	99 (55.0%)
BUN (mg/dl) median (min-max)	60.5 (24.4–118)	66.7 (26.0–118)	60.0 (24.4–105)
Creatinine (mg/dl) median (min-max)	8.9 (1.9–15.0)	6.9 (1.9–12.6)	9.1 (2.7–15.0)
Cholesterol (mg/dl) median (min-max)	162.2 (49–438)	182.1 (117–438)	158 (49–263)
Kt/V median (min-max)	1.6 (0.7–2.3)	1.4 (0.8–2.2)	1.6 (0.7–2.3)
nPCR (g/Kg/day) median (min-max)	1.0 (0.5–1.9)	0.9 (0.5–1.9)	1.04 (0.5–1.8)

Legend *RRT* renal replacemet therapy, *MIS* Malnutrition inflammation score, *SGA* subjective global assessment (A. well nourished, B moderate malnutrition, C severe malnutrition), *HD* hemodialysis, *HDF* hemodiafiltration, *BUN* blood urea nitrogen (predialysis), *Kt/V* dialysis efficiency index, according to Daugirdas 2 formula, *nPCR* normalised protein catabolic rate

None of the differences is statistically significant

## Abbreviations

BMI: Body mass index
BUN: Blood urea nitrogen
CKD: Chronic kidney disease
CRP: C-reactive protein
HD: Hemodialysis
HDF: Hemodiafiltration
Kt/V: Formula for dialysis efficiency
MIS: Malnutrition inflammation score
nPCR: Normalised protein catabolic rate
PTH: Parathyroid hormone
RRT: Renal replacement therapy
SGA: Subjective global assessment
